# New Approach in the Management of Vertical Root Fracture with the Help of Biodentine and CBCT

**DOI:** 10.1155/2020/2806324

**Published:** 2020-09-14

**Authors:** H. C. Baranwal, Nidhi Singh, Neeraj Kumar, Riya Garg, Jyoti Yadav, Richik Tripathi

**Affiliations:** Faculty of Dental Sciences, IMS, BHU, Varanasi, India

## Abstract

**Introduction:**

Vertical root fracture is a big challenge to dentists for its diagnosis and treatment. As the tooth presents poor prognosis, the most common treatment modality is extraction. One can save the tooth from extraction by the reconstruction of fracture fragments followed by intentional reimplantation. New treatment options have arrived for healing of the fracture site by hard tissue barrier formation with the help of bioceramic materials. *Case Report*. The present case report describes successful management of complete vertical root fracture of a maxillary left central incisor by trauma in a 35-year-old male with the help of Biodentine, a bonding agent, a fiber post, and dual-cure resin cement without tooth extraction, followed by all ceramic crowns. Up to two years of follow-up, there is no problem in the tooth, and radiographically, there is no radiolucency along the fracture line. Periodontal status and probing depth were within a normal physiological limit.

**Conclusion:**

Functional and aesthetic outcomes are achieved by combined therapy in the present case report.

## 1. Introduction

Vertical root fracture is defined as a longitudinally oriented fracture of the root, extending from the root canal to the periodontium [1]. Vertical root fracture is one of the most common causes of extraction [2]. Prevalence of vertical root fracture in permanent teeth was 3 to 5% in retrospective studies with etiology being multifactorial [3, 4]. The incidence of VRF is more commonly associated with endodontically than nonendodontically treated teeth [5]. In the vital tooth, physical trauma is the most common reason for tooth/root fracture. In anterior teeth, the line of spread of fracture is in the buccolingual direction [6]. VRF presents male predilection due to their stronger masticatory forces, high attrition, habitual hard food chewing, and less pliable supporting bone [7]. VRF repair using MTA has been already approved [8–11]. But it is difficult to handle; has long setting time, discoloration, and lower compressive and flexural strength than a dentine; and is high-cost [12–15]. Due to this, over the last years, several calcium silicate cements comparable to MTA have been presented, in which Biodentine (Septodont) is one of them. It is a calcium silicate-based cement which was used primarily as a substitute of dentine below resin composite filling material [16]. In contrast to MTA, Biodentine exhibits short setting time of about 15 min [15, 17] and appreciable mechanical features. It is a material which can set off periodontal regeneration and/or restoration. It has beneficial properties in the periodontium regarding biologic reaction of the cells. It is also called “bioactive cement” [18] because of the upregulating action on the activity of osteoblasts and periodontal cells.

Unfortunately, until today, no valid treatment option can be recommended to preserve teeth with VRF. In spite of that, the present case report used Biodentine as a new treatment option for a maxillary incisor with VRF. Using this material in the fractured tooth could be kept in situ for an observation period of two years, and the subject is free of any complaints until now.

## 2. Case Presentation

**Figure 1:**
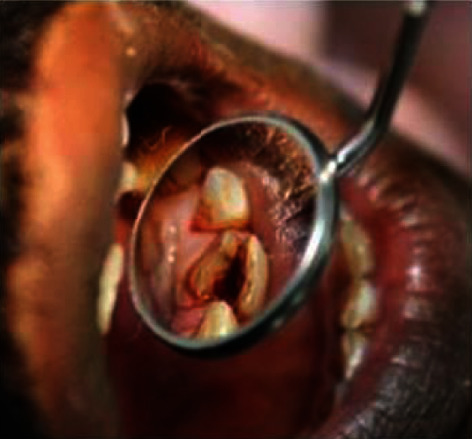
Preop photograph.

**Figure 2:**
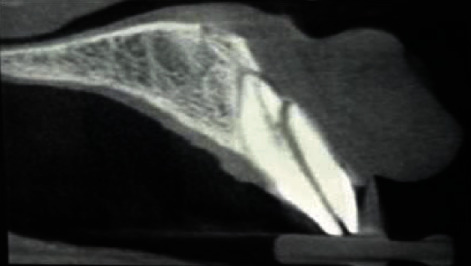
Preop CBCT.

**Figure 3:**
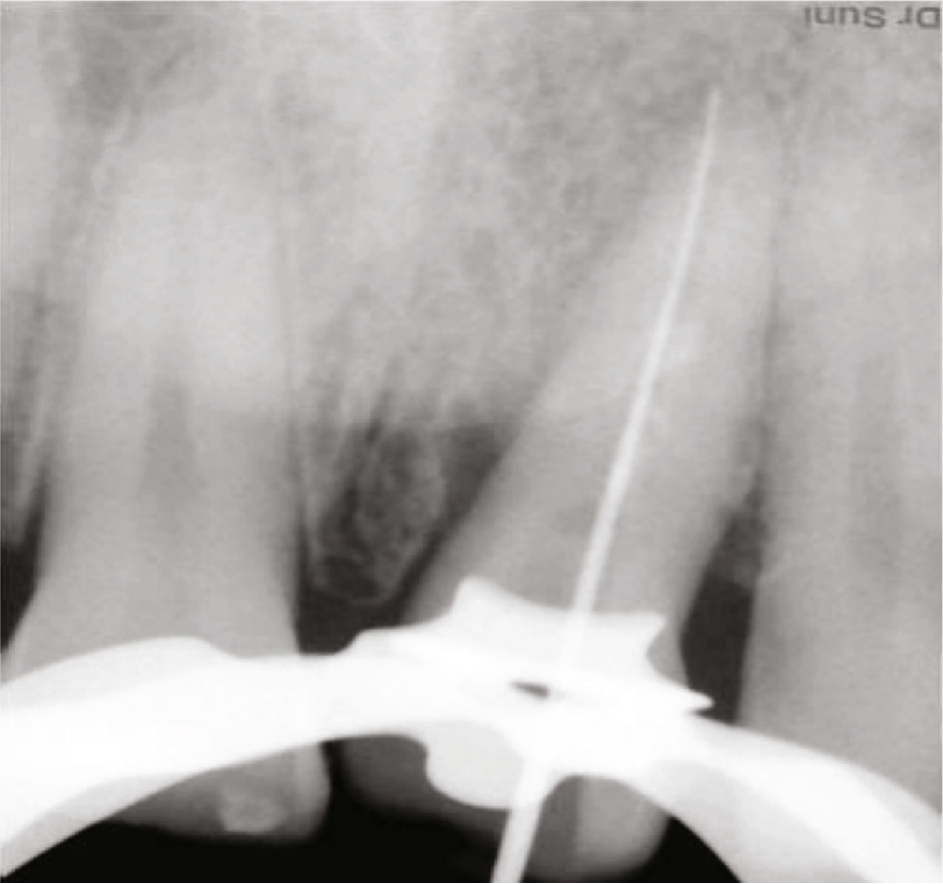
Working length determined.

**Figure 4:**
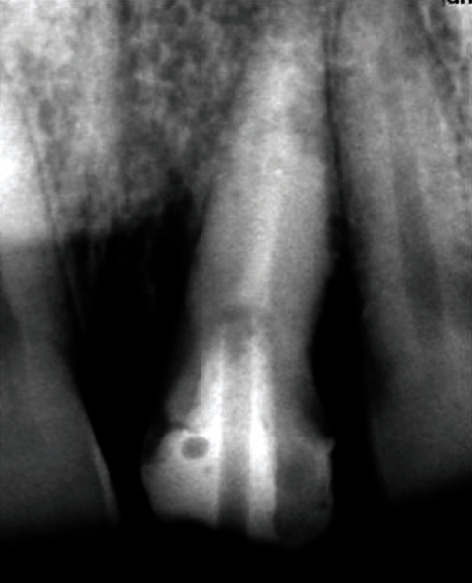
After postcementation.

**Figure 5:**
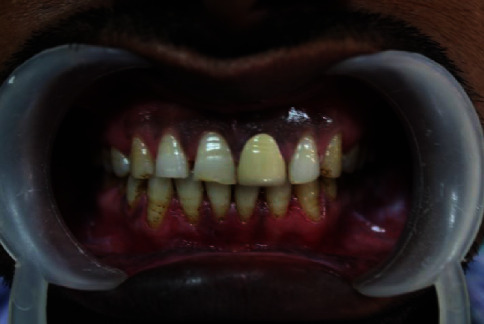
Postop photograph.

**Figure 6:**
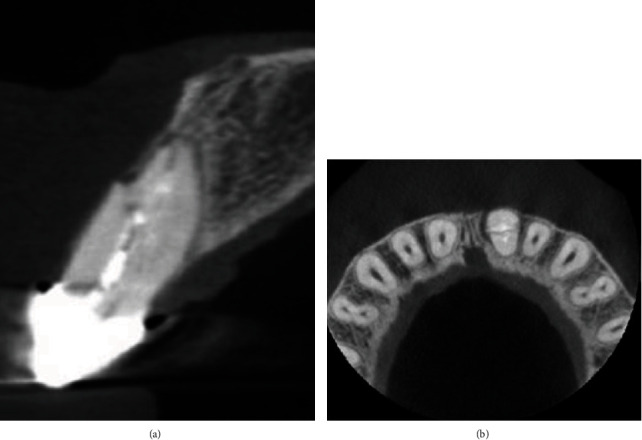
(a) After 6 months of follow-up on CBCT (axial view). (b) After 6 months on CBCT (sagittal view).

**Figure 7:**
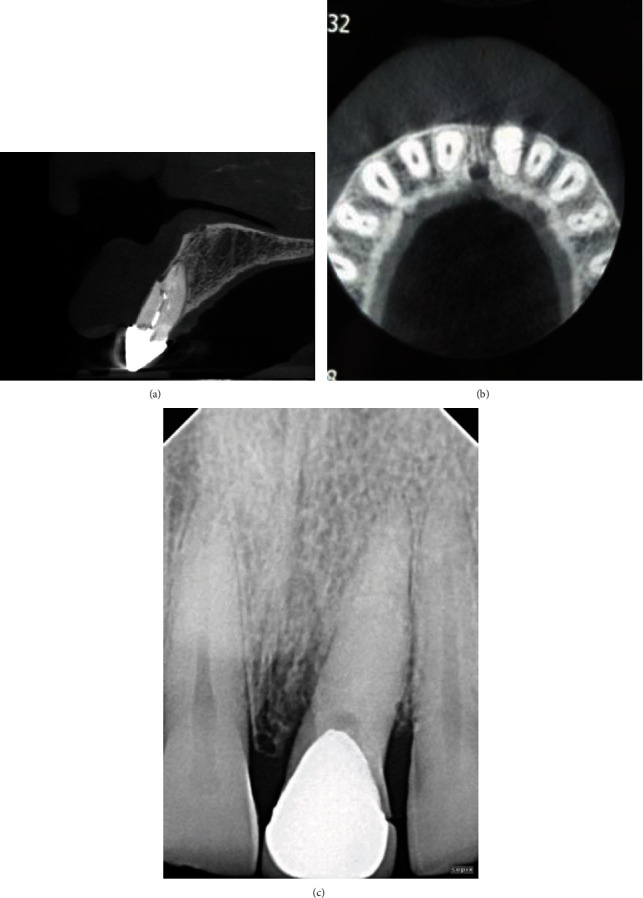
(a) After 1 year of follow-up (axial view). (b) One year of follow-up (sagittal view). (c) One year of follow-up on IOPAR.

**Figure 8:**
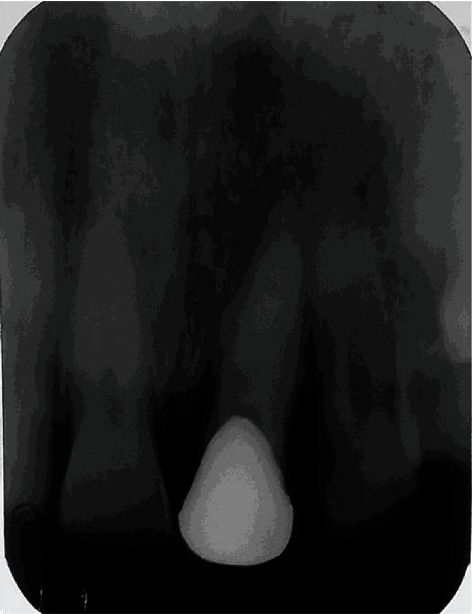
Two years of follow-up.

**Figure 9:**
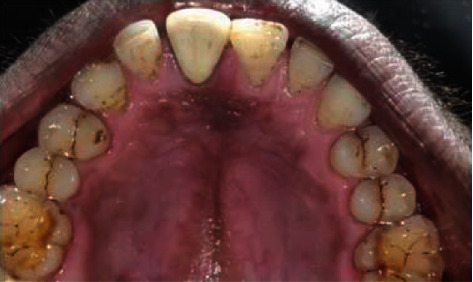
Palatal view photograph after 2 years.

A 35-year-old male patient came to the Department of Conservative Dentistry and Endodontics, IMS, BHU, complaining of a fracture and pain along with cold and heat sensation in the upper left front tooth 8 days back. The patient had a history of trauma due to falling down at home 8 days back. On clinical intraoral examination, a horizontal fracture line was seen in tooth no. 21 (Figure 1). Fractured tooth no. 21 was tender on percussion with a normal pocket depth. A pulp sensitivity test showed exaggerated response to a cold and heat test. On examination by CBCT, a vertical fracture line was shown mesiodistally along the root canal space to the apical third of the root directed labially (Figure 2). In a literature review, the treatment of complete vertical root fracture is the extraction of the tooth followed by dental prosthesis or implant or joining the separated fragments by different types of bonding system, laser or bioceramic cements, and reimplantation with a good success rate. None of these treatment options was accepted by the patient (because of fear of tooth extraction). To save the tooth, a new approach was planned to join the fragments without extraction of the tooth. Written informed consent was obtained, and the patient was scheduled for treatment on the next day after explaining all treatment plans. Before starting the root canal treatment, both separated fragments in the clinical crown region was bonded with a universal dentin bonding (Scotchbond Universal Adhesive 3M ESPE) agent and cured for 20 seconds, and then, flowable composite resin was used (Filtek Z 350xt 3M ESPE) and lightly cured. Firstly, isolation was done with a rubber dam (Hygenic dental dam) with local anesthesia administration (lignocaine, 1 : 150000) before starting the procedure. A proper access opening was done with endoaccess bur. Working length was established with a no. 15 k-file and confirmed by IOPA X-ray (Figure 3). Chemomechanical preparation was done using an up to no. 40 k-file as a master apical file. Irrigation was done by using normal saline and 0.5% NaOCl (Septodont Parcan) during CMP, and the final irrigation was done by using 5 ml 3% NaOCl and 5 ml 17% EDTA (Maarc Endo-L) followed by normal saline. After drying the root canal with a paper point, Biodentine (Septodont) was placed with the help of a Lentulo spiral in the apical part of the root canal, all fracture parts of the line and root canal space up to the intraosseous region. After 24 hours, postpreparation was done by using corresponding drill no. 2 (Tenax fiber post drill) and applying a bonding agent, and dual-cure resin cement (Kerr Maxcem Elite self-adhesive resin cement) was filled in the prepared post space; a no. 2 glass fiber post (Tenax fiber post, Coltene) was inserted and lightly cured for 40 seconds (Figure 4). After core buildup and reduction, impression was taken and the model was prepared. The cast was sent to the dental lab for all ceramic crowns on the same appointment. The crown was luted with the help of resin cement on the next visit (Figure 5). The patient was fully satisfied and happy with treatments even though slight pain and tenderness persist up to 30 days. After 30 days, all clinical signs and symptoms disappeared. The patient was recalled for a follow-up after 6 months (Figures 6(a) and 6(b)), 12 months (Figures 7(a)–7(c)), and 2 years (Figure 8) to check the progress of hard tissue formation on the fracture line by CBCT and X-ray. All periodontal conditions were within a normal limit after 24 months (Figure 9).

## 3. Discussion

Most of the vertical root fractures occur in the root canal-treated tooth and due to traumatic injury. There are very few choices of treatment for complete VRF in the anterior teeth due to traumatic injuries, due to the fact that complication naturally leads to extraction in most of cases. In the past, various successful treatment modalities in the anterior teeth have been suggested by uniting the fracture line by different adhesive techniques like dual-cure resin cement [19], 4 META/MMA/TBB [20], CO_2_ laser, Nd:YAG laser [21], MTA [22], and Biodentine [23] followed by reimplantation of the tooth.

The main factors which lead to extraction are bacterial infection in the fracture line and the resorption of a nearby alveolar bone [24]. So, especially in a vital tooth where there is no alveolar resorption and bacterial infection, a bioceramic material acts as an antibacterial and prevents the alveolar resorption and induces hard tissue deposition. The distance between separated fragments and the position and the extent of the fracture are also important features to determine the treatment modality. Erdem et al. [25] and Kusgoz et al. [26] in 2009 reported that MTA has the ability to seal the repair of horizontal root fracture.

Like MTA, Biodentine is also established for the management of horizontal root fracture, perforation repair, and external root resorption cases. Biodentine is biocompatible and has lesser setting time (15 minutes), and its Vickers hardness is 60 HV which was similar to that of dentin while the microhardness for ProRoot MTA was 40 HV [27]. Biodentine is available in the form of powder and liquid. In the powder form, it contains tricalcium silicate, dicalcium silicate, calcium carbonate, and zirconium dioxide. In the liquid form, it contains calcium chloride in aqueous solution with an admixture of polycarboxylate. Biodentine has greater resistance to hydrolysis during setting and does not dissolve in saliva [15, 17, 28]. Biodentine releases a higher amount of silicon and calcium in comparison to MTA. Silicon helps in bone calcification and stimulates bone growth and mineralization of the dentin [29]. Biodentine develops a calcium- or phosphate-rich hydroxyl apatite-like surface in the presence of body fluid. This surface helps in cell attachment and proliferation of the periodontal ligament [30, 31]. Biodentine might have great advantages when used as a canal obturation material because of its bioactive and physiochemical properties. Biodentine like MTA also provides an effective seal against dentin and cementum and promotes biological repair and regeneration of the periodontal ligament [32, 33]. So Biodentine may be a good choice for VRF without extraoral attachment of fragments and reimplantation. Biodentine was first used to fill the fracture line in prepared incomplete VRF in a maxillary central incisor extending through the buccal surface from the top of the coronal portion to the apex after careful removal and reimplantation, and after 24 months of follow-up, no pathosis was observed [23]. So, Biodentine was used in this case to fill in the prepared root canal for joining and healing the fracture fragments with good success up to two years of follow-up. A post and core may help in the prevention of coronal fracture, when the remaining coronal tooth structure is very thin after tooth preparation [5]. Root strength can also be improved by removing a smear layer. Chelating agents also induced an osteoinductive effect on hard tissue formation by releasing TGF beta1. CBCT can be valuable for the early detection of vertical root fracture in both endodontically and nonendodontically teeth.

## 4. Conclusion

Functional and aesthetic outcomes following treatment are achieved by a combined therapy in the present case report. A long regular follow-up is required to evaluate the success and to do the necessary alternation in the suggested treatment protocol if needed.
